# Oxidant Trade-Offs in Immunity: An Experimental Test in a Lizard

**DOI:** 10.1371/journal.pone.0126155

**Published:** 2015-05-04

**Authors:** Michael Tobler, Cissy Ballen, Mo Healey, Mark Wilson, Mats Olsson

**Affiliations:** 1 University of Sydney, School of Biological Sciences, Sydney, NSW, Australia; 2 Department of Biology, Lund University, Ecology Building, SE-223 62, Lund, Sweden; 3 University of Wollongong, School of Biological Sciences, Wollongong, NSW, Australia; CNRS, FRANCE

## Abstract

Immune system functioning and maintenance entails costs which may limit investment into other processes such as reproduction. Yet, the proximate mechanisms and ‘currencies’ mediating the costs of immune responses remain elusive. In vertebrates, up-regulation of the innate immune system is associated with rapid phagocytic production of pro-oxidant molecules (so-called ‘oxidative burst’ responses). Oxidative burst responses are intended to eliminate pathogens but may also constitute an immunopathological risk as they may induce oxidative damage to self cells. To minimize the risk of infection and, at the same time, damage to self, oxidative burst activity must be carefully balanced. The current levels of pro- and antioxidants (i.e. the individual oxidative state) is likely to be a critical factor affecting this balance, but this has not yet been evaluated. Here, we perform an experiment on wild-caught painted dragon lizards (*Ctenophorus pictus*) to examine how the strength of immune-stimulated oxidative burst responses of phagocytes in whole blood relates to individual oxidative status under control conditions and during an *in vivo* immune challenge with *Escherichia coli* lipopolysaccharide (LPS). Under control conditions, oxidative burst responses were not predicted by the oxidative status of the lizards. LPS-injected individuals showed a strong increase in pro-oxidant levels and a strong decrease in antioxidant levels compared to control individuals demonstrating a shift in the pro-/antioxidant balance. Oxidative burst responses in LPS-injected lizards were positively related to post-challenge extracellular pro-oxidants (reflecting the level of cell activation) and negatively related to pre-challenge levels of mitochondrial superoxide (suggesting an immunoregulatory effect of this pro-oxidant). LPS-challenged males had higher oxidative burst responses than females, and in females oxidative burst responses seemed to depend more strongly on antioxidant status than in males. Our results confirm the idea that oxidative state may constrain the activity of the innate immune system. These constraints may have important consequences for the way selection acts on pro-oxidant generating processes.

## Introduction

A well-functioning immune system is a prerequisite for animals to protect themselves against a multitude of parasites and pathogens. However, maintenance and activation of the immune system is costly. Energy or nutrients invested in immune system functioning must be traded-off against investment into other processes or activities such as growth and reproduction (reviewed in e.g. [1–3]). Moreover, immune system activation also entails the risk of immunopathology when the organism’s immune system causes damage or death to its own cells [4–6]. Hence, up-regulation of the immune system and effective clearing of an infection must also be traded-off against the risk of immunopathological responses which may have wide-ranging consequences for survival and fitness [7].

We still know relatively little about the proximate mechanisms and ‘currencies’ mediating the costs of immune responses. One specific immunopathological cost that has recently gained interest is oxidative damage that can be inflicted through the innate immune system [5,8–10]. Immune cells such as phagocytes and lymphocytes possess specific enzymes and enzyme complexes (e.g. the nicotinamide adenine dinucleotide phosphate (NADPH) oxidase enzyme complex or the nitric oxide (NO) synthase) that can rapidly produce large amounts of reactive oxygen and nitrogen species (hereafter collectively termed reactive species (RS)) [11]. NADPH oxidase in particular can produce massive amounts of RS within a very short time—a process also known as respiratory or oxidative ‘burst’ [9,12]. RS are highly reactive chemical molecules that contain one or more unpaired electrons and, hence, have a high capacity to oxidize other biomolecules. RS produced by immune cells contribute in a variety of ways to the killing of bacteria and other microbes [11,13]. Due to their cytotoxic character, RS can directly contribute to the degradation of the pathogen. Moreover, RS act as immuno-modulatory signal molecules coordinating the migration of the immune cells to the site of infection, and promote the retention of immune cells at that site [11]. Consequently, RS production by immune cells constitutes an important part of the innate immune system. However, increased production of RS through the immune system may also be costly for the host as it may not only result in damage to pathogens but also in damage to own cells. Hence, the risk of infection must be traded off against the risk of oxidative damage to self.

The risk of damage to self is likely to depend on the oxidative state of the individual, i.e. on the concurrent ‘RS-load’ and the levels of antioxidant protection. Under neutral circumstances, inherent RS-production is mainly determined by mitochondrial respiration [14]; during oxidative phosphorylation (i.e. the process that produces chemical energy (ATP)), a small percentage of oxygen is converted into RS instead of water by the electron transport chain located in the mitochondrial membrane [12]. All cells that possess mitochondria will constantly leak RS through respiration which, in excess, may cause oxidative damage. To minimize and protect tissues and cells from oxidative damage, many organisms possess a sophisticated system of endogenous and diet derived antioxidant defences (e.g. specific enzymes such as superoxide dismutase or catalase and dietary antioxidants such as carotenoids or vitamin E [12]). Depending on the availability and activity of these defences, it is possible that different RS-generating processes may constrain each other [9]. For example, high mitochondrial RS production may limit the pro-oxidant activity of the immune system. In a previous study, we found that systemic levels of mitochondrial superoxide (SO), the primary RS produced in cell respiration, are negatively correlated with the strength of the immune response towards the mitogen phytohaemagglutinin (PHA) in male painted dragon lizards (*Ctenophorus pictus*; Peters 1866) [15]. PHA induces local inflammation and associated infiltration of many immune cell types such as lymphocytes and macrophages [16]. Potentially, mitochondrial SO levels act immunoregulatory, mediating the trade-off between immunoinvestments and other processes. That mitochondrial SO production influences innate immune function is corroborated by studies on mutant mice: mice with reduced mitochondrial SO production showed an enhanced inflammatory response [17] whereas mice with elevated mitochondrial SO levels experienced an impaired T-cell development and function [18].

Few studies have aimed to quantify the oxidative burst response in non-model organisms [19–22] and very little is known about how the strength of the oxidative burst response relates to the pro-/antioxidant balance before and during an infection. Such knowledge is important, however, if we want to better understand the role of RS as a cost of immunity and as ‘universal constraints in life-history evolution’ [9]. The purpose of this study was therefore to directly quantify the oxidative burst response in wild-caught painted dragon lizards before and after an immune challenge with *Escherichia coli* lipopolysaccharide (LPS) and to assess how it is related to different baseline estimates of oxidative status. LPS is an endotoxin found in the outer membrane of Gram-negative bacteria. It mimics a bacterial infection and elicits an inflammatory response [23], which involves the activation of phagocytic cells and an increase of RS production through the oxidative burst reaction [12,20]. Given that higher mitochondrial SO levels appear to counteract inflammatory responses in males (see above), we predicted a similar negative relationship between SO levels and the strength of the oxidative burst response (at least in males).

Male and female painted dragons differ in many behavioural and reproductive aspects. Males compete actively for mating opportunity resulting in high levels of aggressive territorial interactions and increased exposure to predators [24,25]. Females, on the other hand, have a more cryptic, sedentary life style which involves frequent basking to maintain appropriate incubation conditions for developing follicles and eggs [25]. Elsewhere, we show that male and female painted dragons also differ in a range of physiological characteristics, including RS production, antioxidant regulation and immune responsiveness [15,26–28]. In the present study, we therefore specifically examined whether measures of oxidative burst and their associations with systemic pro- and antioxidants differ between the sexes. The results of our study offer new insights into individual and sex-specific variation of the oxidative burst response with implications for our general understanding of the costs and benefits of immune system-generated RS production.

## Materials and Methods

### Ethics statement

The study was conducted according to the guidelines of the University of Sydney Animal Care and Ethics Committee. All experimental procedures and protocols were approved by the the University of Sydney Animal Care and Ethics Committee (permit no. L04/10-2010/3/5386) and capture of lizards in the wild was licensed by the National Parks and Wildlife Service, New South Wales, Australia (permit no. SL100352). After the experiments, lizards were released at their original site of capture.

### Animal husbandry and blood sampling

The Australian painted dragon is a small (adult snout-vent length 65–95mm and mass 8–16g) diurnal lizard inhabiting open sandy areas with low vegetation with a range covering central and western New South Wales to Western Australia. It is short-lived (only ca 10% live to a second year), males are strongly territorial and females typically produce two-three clutches in natural populations. The lizards were caught by noose or by hand near Yathong Nature Reserve, New South Wales (145°35’; 32°35’) during the reproductive season (November 2010) and brought back to holding facilities at the University of Sydney. All lizards were kept individually in cages (60×60×50 cm), on a 12:12 h light regime (light:dark), with a spotlight at one end of the cage to allow thermoregulation to the preferred body temperature (36–37°C, M.Olsson unpublished data) and fed crickets and meal worms every second day.

Sample sizes for this study were N = 31 for males, and N = 30 for females. For the purpose of another experiment, about half of the males (N = 15) were implanted subcutaneously with small, empty silastic tubes (i.d. 1.47 mm, o.d. 1.96 mm, Dow Corning, Midland, MI, USA) six weeks prior to the study described here. Empty implants have no measurable effect on any of the assayed blood parameters in these males (no implant versus empty implant males, F<1.3, df = 1,29, p>0.25). We therefore pooled all males in subsequent analyses.

To assay pro- and antioxidant parameters, we collected two blood samples from each lizard with a glass capillary from *vena angularis* (in the corner of the mouth) (Table 1). Because it was not logistically possible to conduct all analyses on fresh blood on the same day, we blood-sampled the lizards in two batches, on two consecutive days. The first blood sample (80 μl) was collected on 1 and 2 February. A second blood sample (40 μl), was collected six days later, on 7 and 8 February. This second sample was collected subsequent to an injection with either lipopolysaccharide (LPS) dissolved in phosphate buffered saline (PBS) or PBS only (see below). A smaller sampling volume was required for the second sample due to the short re-sampling interval and the possible strenuous effects of the immune challenge. The frequencies of males and females did not differ between sampling batches (18 males/15 females versus 13 males/15 females; χ^2^ = 0.40, df = 1, p = 0.53). Similarly, the frequency of LPS- versus PBS-treated lizards did not differ between batches (17 LPS/16 PBS versus 13 LPS/15 PBS; χ^2^ = 0.16, df = 1, p = 0.69).

**Table 1:** Summary of the measures of oxidative physiology collected during the two sampling events.

Measure	pre-challenge^a^ (1 & 2 February)	post-challenge^a^ (7 & 8 February)
Mitochondrial superoxide (mSO)^b^	X	
Unspecific intracellular reactive species (iRS)^b^	X	
Unspecific extracellular reactive species (eRS)^b^	X	X
Peak oxidative burst response (peak OBR)^c^	X	X
Total oxidative burst response (total OBR)^c^	X	X
Total plasma antioxidant levels (plasma AOL)^b^	X	X

^a^: pre-challenge refers to control conditions whereas post-challenge refers to measures after injections of the lizards with either LPS or PBS.

^b^: baseline measures of oxidative status of intra- and extracellular pro-and antioxidants.

^c^: maximum and total phagocytic reactive species production in response to *in vitro* lipopolysaccharide stimulation.

### LPS challenge

In order to compare RS production and antioxidant levels in lizards with and without an activated immune system, we simulated an infection using LPS in a subset of the lizards. On 6 and 7 February, 16 males and 14 females were injected subcutaneously with LPS (2.5μg per g body mass; [29]) from *E*.*coli* (055:B5; Sigma L2880; same strain as used for *in vitro* activation of blood cells in the oxidative burst assay, see below). The other 15 males and 16 females received an injection with vehicle only (PBS). To assess oxidative burst in response to LPS, we used the same study design as Sild & Horak [20] injecting lizards in the evening and collecting a blood sample in the following morning. The time of injection did not differ between sexes or treatment groups (sex: F_1, 59_ = 1.29, p = 0.26, treatment: F_1, 58_ = 0.00, p = 0.98, sex × treatment: F_1, 57_ = 0.00, p = 0.98). The day after the LPS/PBS injection (7 or 8 February), approximately 18 hours (mean ± 1 SE: 18.50 ± 0.15) post injection, the second blood sample was collected. There was no difference in the time interval between injection and blood sampling among sexes or treatment groups (sex: F_1, 59_ = 1.81, p = 0.18, treatment: F_1, 58_ = 0.27, p = 0.60, sex × treatment: F_1, 57_ = 0.05, p = 0.82). Concomitant with blood sampling we also collected measures of body mass (to the nearest 0.01 g) and snout-vent-length (SVL; to the nearest 0.5 mm). For one female (PBS group) we failed to collect a body mass measure during the first sampling event.

### Quantification of blood parameters

The assayed six different blood parameters are listed in Table 1. Five of these measures (mitochondrial superoxide, unspecific intracellular and extracellular RS, peak and total oxidative burst response) required fresh blood for analysis whereas total antioxidant levels were quantified from plasma. The measures of oxidative burst (both peak and total) quantify the RS production of blood cells in response to an *in vitro* stimulation with LPS (see below). The other four measures did not involve *in vitro* stimulation and, hence, are considered baseline estimates of RS production and antioxidant status (Table 1). Measures from the first blood sample relate to the oxidative state of lizards under control conditions (pre-challenge), whereas measures from the second blood sample relate to the oxidative state of lizards which were either injected with LPS or PBS (post-challenge).

### Intracellular RS levels

We used flow cytometry in combination with two probes (MitoSOX Red (MR) and dihydrorhodamine 123 (DHR), Invitrogen) to quantify excess RS produced in blood cells.

MR gives an estimate of mitochondrial superoxide (hereafter *mSO*), the primary RS produced in cell respiration. The probe diffuses into cells and accumulates in the mitochondria where it becomes fluorescent when oxidized by mSO, but not other RS. DHR estimates the total amount of unspecific intracellular RS (hereafter *iRS*) load. It diffuses into cells where it can be oxidized by various RS including singlet oxygen, superoxide, hydrogen peroxide and peroxynitrite. Both probes compete with cellular antioxidants that also neutralize RS. Hence, both measures depend on the rate of RS production as well as on the amount of cellular antioxidants. Higher levels of mSO or iRS indicate a stronger imbalance between RS generation and elimination, i.e. more excess RS. Our previous findings show that measures of mSO and iRS are associated with colour maintenance, immune activation and reproductive investment [15,28,30,31]. Note that due to limited blood volumes, measures of mSO and iRS levels could only be obtained from the first blood sample.

For quantification of mSO and iRS, 20 μl of freshly obtained blood was diluted immediately 1:10 with phosphate buffered saline (PBS; 137 mM NaCl, 2.7 mM KCl, 1.5 mM KH_2_PO_4_, 8 mM Na_2_HPO_4_, pH 7.4) and stored on ice prior to analyses, which were completed within 4 h of sampling. Flow cytometry analyses were conducted according to previously specified protocols [30]. Elsewhere, we have shown that measurements of intracellular RS sampled on the same day are consistent (correlation coefficients for separate blood samples from 14 males were r = 0.97 (p<0.0001) for mSO and r = 0.80 (p = 0.0006) for iRS) [30]. Between-batch variation in assay conditions was accounted for by standardising the data by batch, setting means to zero and standard deviations to one before pooling the RS measures for analysis.

### Quantification of extracellular RS production

To obtain measures of extracellular RS production, we used the ABEL cell activation test kit (ABEL-06M; Knight Scientific, Plymouth, UK). This assay makes use of the photoprotein Pholasin, which becomes chemiluminescent upon reaction with RS. Pholasin is a 34-kD protein that is too large to enter cells and the light emitted by Pholasin is, thus, proportional to the amount of excess extracellular pro-oxidants in a blood sample (Pholasin competes with antioxidants and higher levels of luminescence indicate a stronger imbalance between RS generation and elimination). To measure the oxidative burst capacity of phagocytes in lizard blood, cells were stimulated *in vitro* with LPS. LPS activates the NADPH oxidase complex located in phagocytic membranes resulting in a rapid increase of extracellular RS (S1 File; S1 Fig) [20]. We calculated two parameters: i) the peak luminescence or peak oxidative burst response (hereafter *peak OBR*) as a measure of the strength of the phagocytic response (the maximum capacity of the phagocytes to produce RS in response to stimulation) and ii) the cumulative luminescence or total oxidative burst response (hereafter *total OBR*) as a measure of both the strength and the stamina of the phagocytic response (the total amount of RS produced in response to stimulation). The average luminescence prior to the *in vitro* LPS-stimulation gives a baseline estimate of unspecific extracellular RS (hereafter *eRS*) produced by non-stimulated blood cells and reflects the cell activation state at the time of measurement (see S1 File).

To perform the assay, fresh lizard blood (20μL) was refrigerated in heparinized 0.5 mL Eppendorf tubes until analyses (7–10 h post-sampling). Before running the assay, refrigerated blood was incubated for 10 min at 37°C. We followed the protocol described by Sild and Horak [20] slightly modified for our lizard blood samples (see S1 File). All samples were run in duplicates and mean values were used in analyses. For samples run on the same day, interassay repeatabilities of the three measures were R = 0.74 (F_12, 13_ = 6.67, p = 0.0009), R = 0.91 (F_12, 13_ = 23.72, p<0.0001) and R = 0.86 (F_12, 13_ = 12.95, p<0.0001) for eRS, peak OBR and total OBR, respectively. Between-batch variation in assay conditions was accounted for by standardising the data by batch, setting means to zero and standard deviations to one before pooling the data for analysis.

Sample size for the estimate of eRS, peak and total OBR for the first sampling event is reduced by one (LPS-challenged female) due to failed *in vitro* stimulation of the cells and there was not enough fresh blood to re-run the sample. Hence, sample size for these analyses is N = 60.

### Quantification of total plasma antioxidant levels

Lizard blood not used in flow cytometry and cell activation assays was centrifuged at 1000 ***g*** and plasma was stored at -80°C. Plasma antioxidant levels (hereafter *plasma AOL*) were measured using the Abel-41M2 test kit (Knight Scientific Limited, Plymouth, UK). The test measures the capacity of a plasma sample to withstand oxidation by peroxynitrite. Plasma AOL are expressed in vitamin E analogue (6-hydroxy-2,5,7,8-tetramethylchroman-2-carboxylic acid) equivalent units μmol L^-1^ (standard range: 50–800 μmol L^-1^) using the linear regression formula of the standard curve. We performed the assay according to the manufacturer’s instructions but with slightly reduced reagent and sample volumes (see S1 File for details). All samples were run in duplicates. The mean of the duplicate values was used in the statistical analyses. Interassay repeatability for plasma AOL (duplicate samples run on different plates, but on the same day) was R = 0.89 (F_19, 20_ = 16.58, p<0.0001). There was inter-assay variation between samples from the two batches due to differences in assay conditions (see S1 File) and we therefore standardized the antioxidant measures in the same way as the RS measures (see above).

### Statistical analyses

Statistical analyses were performed in SAS System 9.3 for Windows (SAS Institute Inc., Cary, NC, USA). All variables except SVL and iRS levels were log-transformed. The transformed data satisfied assumptions for parametric tests.

Using Pearson correlations (PROC CORR), we first examined whether these baseline estimates (collected prior to the LPS-challenge) were correlated with each other and with body mass or size (SVL). Second, we evaluated whether baseline estimates predicted *in vitro* stimulated phagocytic RS production before the LPS-challenge. For these analyses, we used general linear models (PROC GLM) that included peak OBR or total OBR as response variable, sex as a fixed factor and all four baseline estimates as covariates. Although some of the covariates were correlated, variance inflation factors (range = 1.034–1.517; PROC REG) indicated that multicollinearity was not an issue.

To assess the effect of LPS treatment on baseline estimates (eRS, plasma AOL) and *in vitro* stimulated phagocytic RS production (peak and total OBR), we used general linear models with treatment (LPS or PBS) and sex as fixed factors. The pre-challenge value of the response variable was incorporated in the models as a covariate. Because the estimate of eRS is a strong predictor of OBRs (see results) and because we wanted to assess the increase in RS production relative to the baseline estimate, we also included eRS as an additional covariate in the models on OBRs. However, analyses excluding eRS as a covariate produced qualitatively very similar results.

Subsequently, we investigated the relationship between OBRs and eRS/plasma AOL in immune-challenged lizards only. We expected that activation of the immune system would strengthen or perhaps even change the associations between OBRs and baseline estimates of oxidative status. We used a general linear model with peak or total OBR as response variable, sex (fixed factor), the pre-challenge value of the response variable, eRS and plasma AOL (covariates) as predictor variables.

Finally, we were also interested in whether the baseline estimates of intracellular RS levels prior to the LPS-challenge would predict the oxidative burst responses after the challenge. The rationale was that the priming effect of (or the responsiveness toward) LPS may limited by the inherent RS-profile of the individual (see introduction). Hence, individuals with (consistently) high inherent RS production may be less capable of sustaining strong OBRs with potentially cytotoxic effects. To test this, we run models for both peak and total OBR that included the two pre-challenge measures of intracellular RS production (mSO, iRS) and the pre-challenge value of the response variable as predictor variables. As in the previous models, we also included baseline eRS as an additional covariate, and sex as a fixed factor.

We consistently started with full models, which included the main effects, the two-way interaction between fixed factors (when applicable) and all two-way interactions between the fixed factor(s) and the covariates. To minimize the risk of type I errors, we first tested the full model against a null model (with intercept only) using a likelihood ratio test and only when the full model was significant we proceeded with model simplification [32]. Full models were simplified using backward elimination at a significance level of 0.05. Elimination was performed in a hierarchical manner so that main effects would only be removed if they were not involved in an interaction. Unless noted otherwise, we report results from the final models following backward elimination. Effect sizes from minimal models are reported as eta-squared (η^2^): the ratio of the effect sum of squares over the total sum of squares [33,34]. Full models are reported in S1 Tables.

## Results

### Correlations among pre-challenge oxidative parameters and body size

First, we examined whether baseline estimates of RS production and plasma antioxidants prior to the challenge were correlated with each other. Only two of the estimates were moderately correlated (Table 2). Levels of mSO were positively correlated with levels of eRS and plasma AOL were negatively correlated with eRS. None of the baseline oxidative parameters were correlated with body mass or size (SVL) (Table 2).

**Table 2:** Pearson correlations between different baseline estimates of blood pro- and antioxidant parameters, body size (snout-vent-length) and body mass in painted dragon lizards.

	iRS		mSO		plasma AOL		SVL		body mass	
	r	P	r	P	r	P	r	P	r	P
**eRS**	-0.05	0.69(60)	0.27	0.039(60)	-0.52	<0.0001(60)	-0.01	0.93(60)	-0.13	0.31(59)
**iRS**			0.11	0.41(61)	0.04	0.76(61)	0.08	0.53(61)	-0.06	0.68(60)
**mSO**					-0.09	0.47(61)	0.03	0.81(61)	0.14	0.29(60)
**plasma AOL**							-0.02	0.90(61)	0.19	0.15(60)
**SVL**									0.62	<0.0001(60)

Abbreviations: mSO = mitochondrial superoxide; iRS = unspecific intracellular reactive species; eRS = unspecific extracellular reactive species; plasma AOL = plasma antioxidant levels.

Sample sizes are given in parentheses.

### Pre-challenge oxidative burst responses

The measure of eRS was the sole predictor of *in vitro* induced pre-challenge OBRs (peak OBR: slope ±1SE: 0.580±0.178, F_1, 50_ = 15.08, p = 0.0003, η^2^ = 0.32 (full model statistics); total OBR: slope: 0.634±0.102, F_1, 58_ = 38.98, p<0.0001, η^2^ = 0.40). Neither intracellular RS (mSO, iRS) nor plasma AOL predicted pre-challenge OBRs when entered in the same model (all effects p>0.20).

Sex did not influence pre-challenge peak or total OBRs (main effect and all interactions, p>0.25 in all cases).

### Effect of LPS treatment

**Fig 1:**
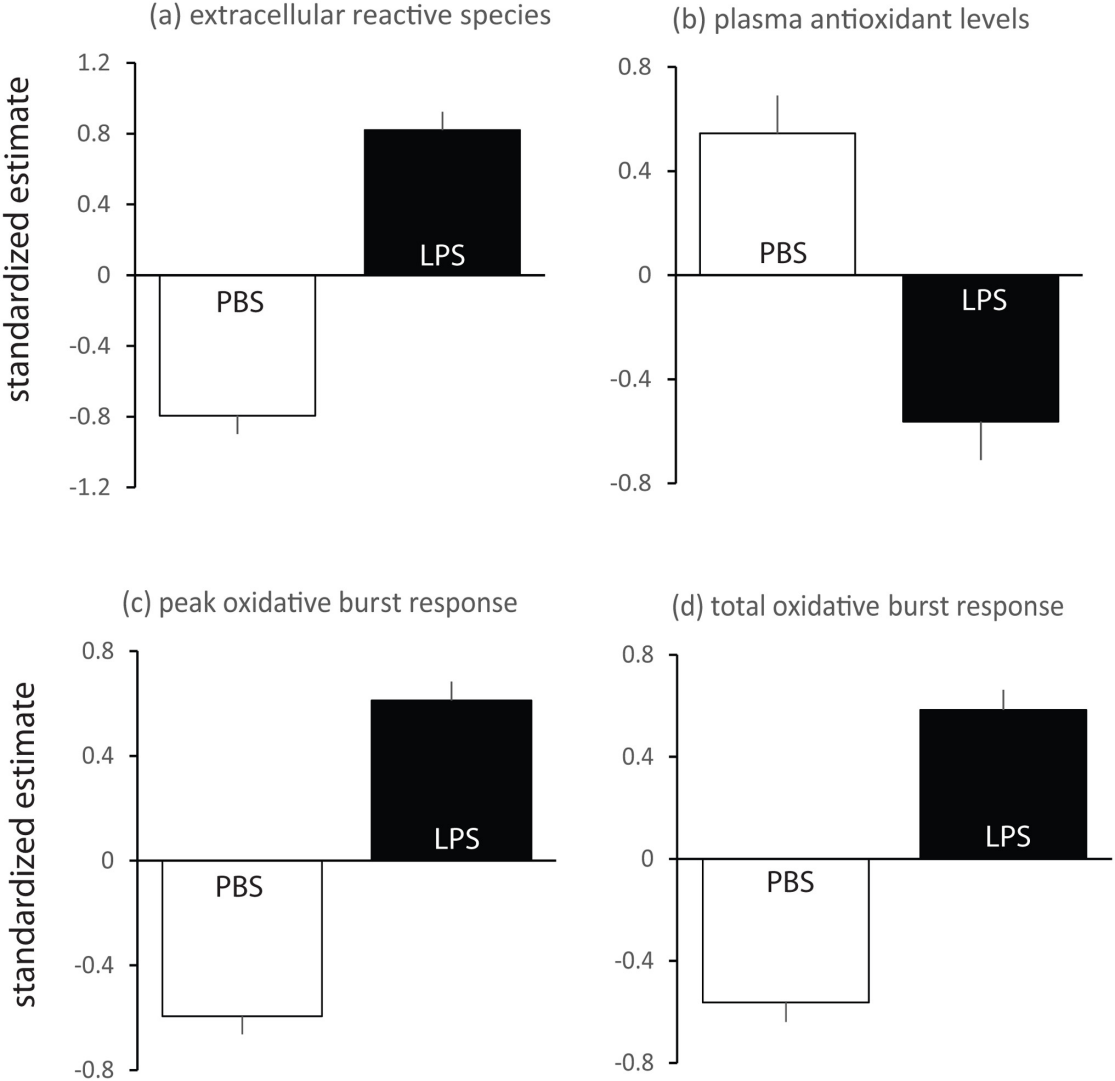
Effects of treatment (LPS or PBS injection) on pro- and antioxidant estimates. Differences in baseline estimates (extracellular RS and plasma antioxidants; a,b) are depicted in the upper panel and differences in *in vitro* stimulated phagocytic RS production (peak and total oxidative burst responses; c,d) are depicted in the lower panel. Parameter estimates are standardized by assay run (see methods); shown are LSmeans ± 1SE. For the comparison of plasma antioxidant levels, samples sizes are N = 31 for the LPS-treated lizards and N = 30 for the PBS-treated lizards. For the other three comparisons sample sizes are N = 30 for both groups (see also main text).

LPS treatment had a strong effect on both eRS and plasma AOL. Levels of eRS were significantly elevated and plasma AOL levels were significantly decreased in LPS-treated as compared to control lizards (Table 3, Fig 1). Sex did not affect eRS and plasma AOL (main effect and interactions all p>0.09).

**Table 3:** Effects of sex and treatment (LPS or PBS injection) on pro- and antioxidant estimates.

Predictor^a^	d.f.	F-value	η^2^ ^b^	P-value
*unspecific extracellular reactive species*				
treatment	1,59	121.62	0.67	<0.0001
*plasma antioxidant levels*				
treatment	1,58	28.91	0.32	<0.0001
pre-challenge plasma AOL	1,58	4.95	0.05	0.03
*peak oxidative burst response*				
sex	1,55	7.90	0.01	0.007
treatment	1,55	96.42	0.11	<0.0001
pre-challenge peak OBR	1,55	15.40	0.02	0.0002
eRS	1,55	40.96	0.05	<0.0001
*total oxidative burst response*				
sex	1,55	7.55	0.01	0.008
treatment	1,55	71.27	0.10	<0.0001
pre-challenge total OBR	1,55	10.02	0.01	0.003
eRS	1,55	36.55	0.05	<0.0001

Presented are reduced models after backward elimination (full models are reported in S1 Tables).

^**a**^ **:** abbreviations: eRS = unspecific extracellular reactive species, plasma AOL = plasma antioxidant levels, peak/total OBR = peak and total oxidative burst response.

^**b**^ **:** effect size: the ratio of the effect (type III) sum of squares over the total sum of squares.

**Fig 2:**
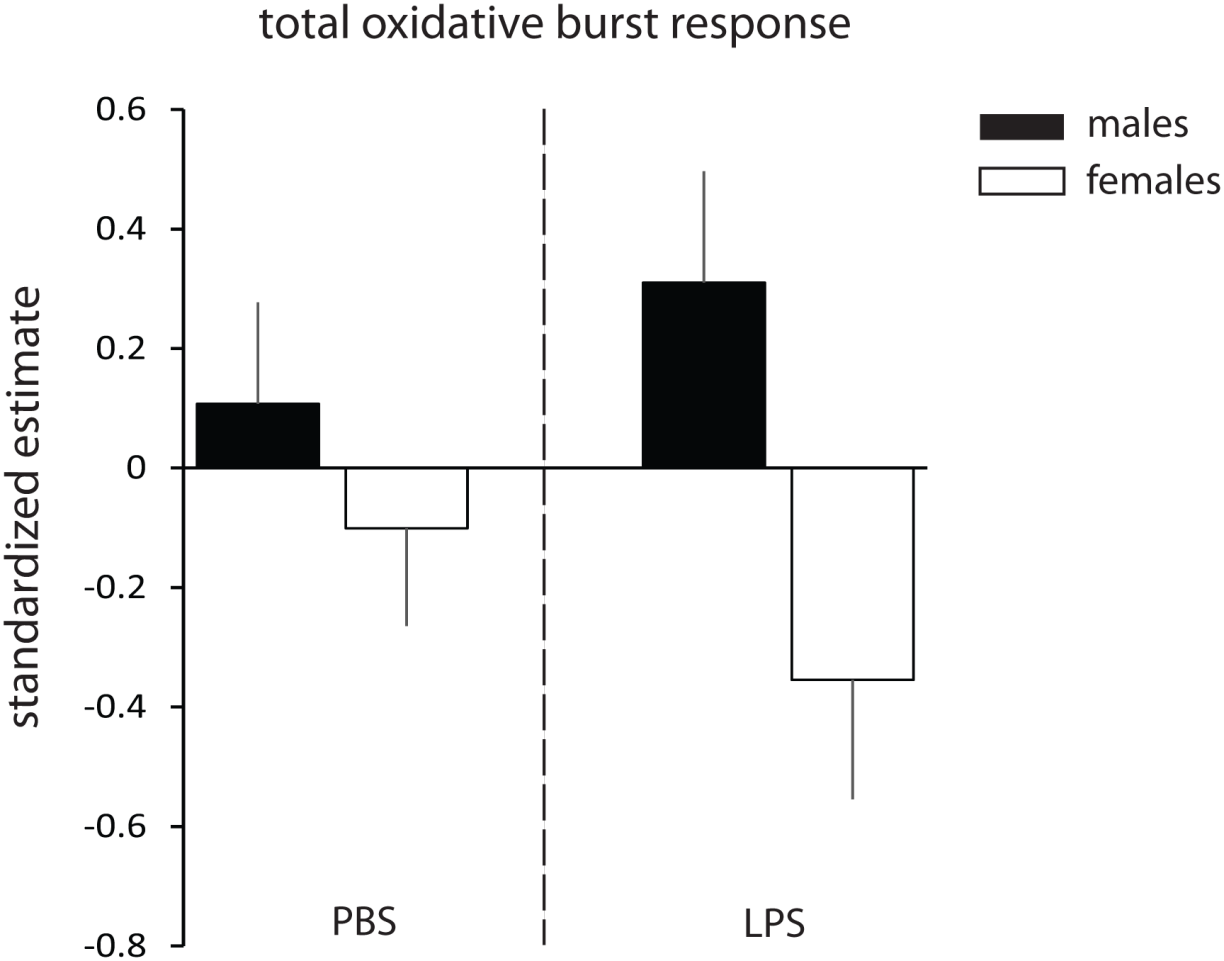
Sex differences in total oxidative burst responses. Depicted are LSmeans ± 1SE for PBS- and LPS-treated males and females. Parameter estimates are standardized by assay run (see methods). Samples sizes are 29 (16 males and 13 females) for LPS-treated lizards and 31 (16 females and 15 males) for PBS-treated lizards.

Blood cells from LPS-treated lizards produced stronger OBRs than those from PBS-treated lizards (Table 3, Fig 1). Peak and total OBRs differed significantly between sexes with males showing stronger responses than females (Table 3). The significant sex difference was driven by the difference between LPS-challenged males and females (F>4.90, df>25, p<0.05 for peak and total OBR; Fig 2). Pre-challenge OBRs (collected 5 days before LPS-injection) and eRS were also predictors of post-challenge OBRs. Higher levels of eRS and pre-challenge OBRs were generally associated with stronger post-challenge OBRs. Associations between different estimates of oxidative status/OBR did not differ with respect to treatment or sex (all interactions p>0.08).

**Fig 3:**
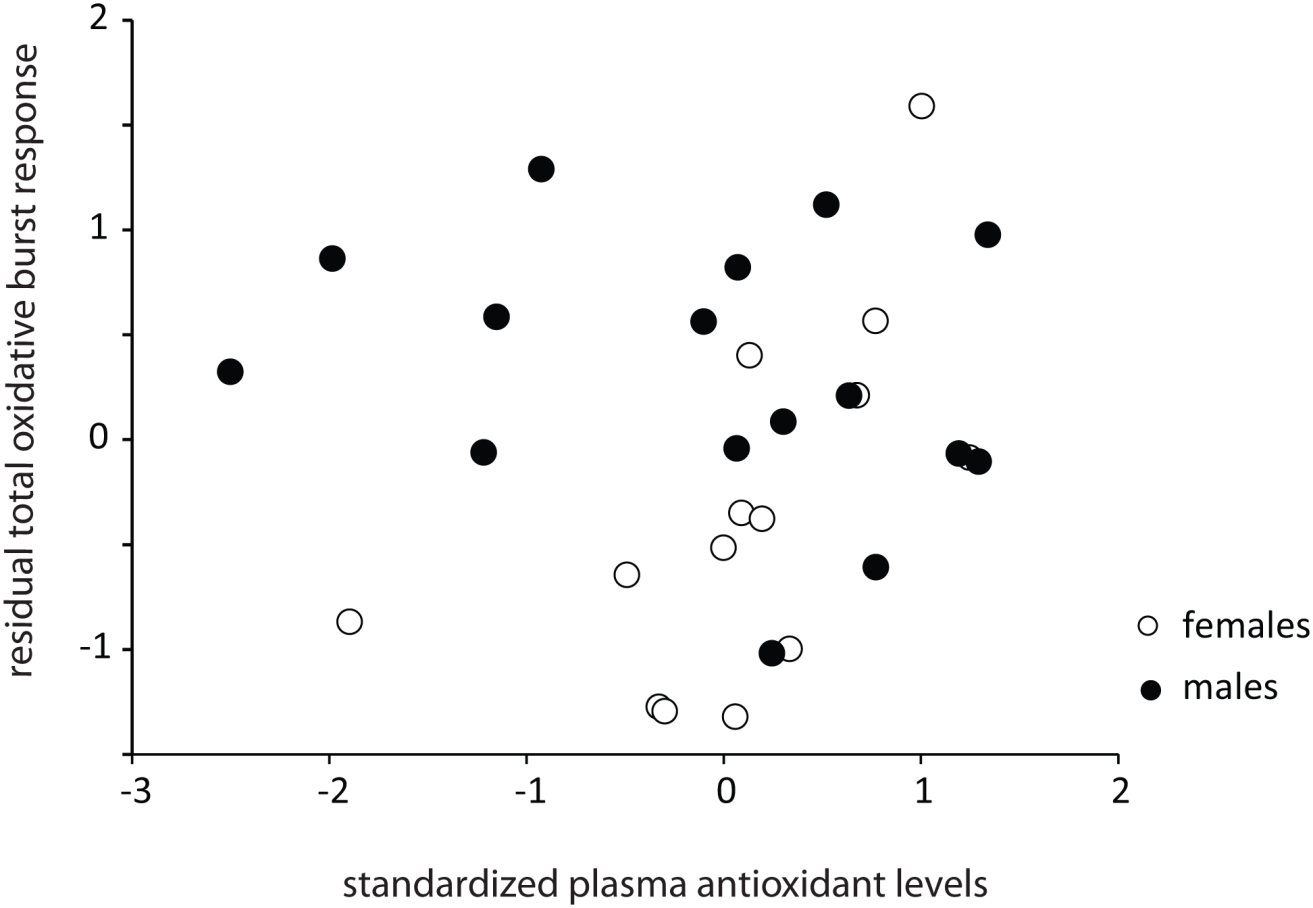
Relationships between plasma antioxidant levels and total oxidative burst responses in LPS-challenged lizards only. The y-axis reflects the residuals of the model (accounting for variation in extracellular RS) with plasma antioxidant levels excluded as a predictor. Females (open circles): N = 14, males (filled circles): N = 16.

OBRs of (primed) blood cells (from LPS-challenged lizards only) showed significant associations with both eRS and plasma AOL. Levels of eRS were positively associated with both peak and total OBRs (peak OBR: F_1, 25_ = 14.68, p = 0.0008, η^2^ = 0.32; total OBR: F_1, 25_ = 20.37, p = 0.0001, η^2^ = 0.24), and this relationship was not influenced by sex (sex×eRS interaction, p>0.25 in both cases). However, the association between OBRs and plasma AOL was a significantly influenced by sex (peak OBR: sex×plasma AOL, F_1, 25_ = 5.32, p = 0.03, η^2^ = 0.08; total OBR: sex×plasma AOL, F_1, 25_ = 7.05, p = 0.014, η^2^ = 0.12). There was a positive relationship between peak/total OBR and plasma AOL in females (slope ±1SE: peak OBR: 0.61±0.24; total OBR: 0.64±0.25) and a negative relationship in males (slope ±1SE: peak OBR: -0.06±0.17; total OBR: -0.15±0.18) (Fig 3).

**Fig 4:**
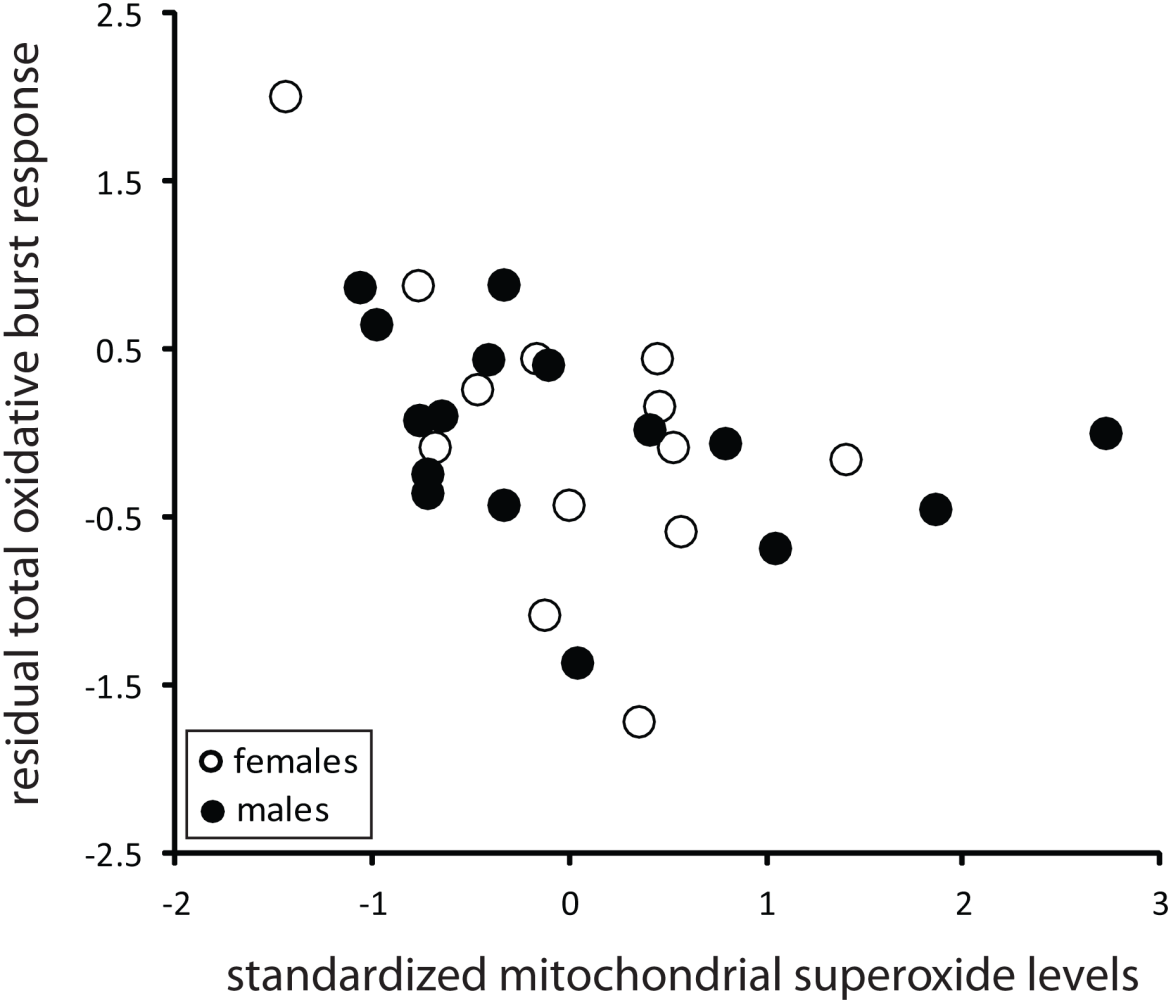
Relationship between total oxidative burst response and mitochondrial superoxide levels in LPS-treated lizards. Mitochondrial superoxide levels are standardized by assay run (see methods). The y-axis reflects the residuals of the model (Table 3) with superoxide levels excluded as a predictor. Females (open circles): N = 13, males (filled circles): N = 16.

Finally, we examined whether baseline estimates of intracellular RS production (mSO, iRS) would predict peak and total OBRs of LPS-challenged lizards. For the peak OBR only sex and eRS remained as significant predictors in the final model (Table 4). However, total OBR was significantly negatively associated with mSO levels (Table 4, Fig 4). In addition, total OBR was affected by an interaction between sex and eRS (Table 4). Although the association was positive in both sexes, the slope was steeper in males than in females (0.838±0.162 versus 0.109±0.205). Pre-challenge iRS levels did not predict total OBR (main effects and interaction, p>0.5).

**Table 4:** Predictors of peak and total oxidative burst responses in LPS-treated lizards only.

Predictor^a^	d.f.	F-value	η^2^ ^b^	P-value
*peak oxidative burst response*				
sex	1,27	4.96	0.09	0.035
eRS	1,27	23.15	0.43	<0.0001
*total oxidative burst response*				
sex	1,23	6.86	0.09	0.015
eRS	1,23	14.81	0.21	0.008
sex × eRS	1,23	7.06	0.09	0.014
mSO	1,23	7.83	0.10	0.010
rre-challenge total OBR	1,23	5.04	0.06	0.035

Presented are reduced models after backward elimination (full models are reported in S1 Tables).

^**a**^ **:** abbreviations: eRS = unspecific extracellular reactive species, mSO = mitochondrial superoxide, total OBR = total oxidative burst response.

^**b**^ **:** effect size: the ratio of the effect (type III) sum of squares over the total sum of squares.

## Discussion

Current theory states that elevated RS production due to immune system activation may constitute a cost, reducing the level of antioxidant protection and increasing the risk of oxidative damage (see introduction). Consistent with this, we found elevated levels of eRS and lowered plasma AOL in lizards that were challenged with the immuno-stimulant LPS.

Furthermore, we show that LPS-challenged lizards exhibit stronger OBRs indicating higher NADPH oxidase activity. Very little is known about the effect of immune system activation on measures of oxidative status in reptiles. Meylan et al. [35] showed that a challenge of pregnant female common lizards (*Lacerta vivipara*) with sheep red blood cells increased activity of the superoxide dismutase enzyme, but only in corpulent females. This finding is consistent with the idea that superoxide dismutase enzyme activity is up-regulated in response to increased RS production through oxidative burst. However, other antioxidant enzymes (catalase, glutathione peroxidase) were not affected by the immune challenge [35]. The results from our study are congruent with those reported in birds and bats. Several studies have shown that immune challenge reduces antioxidant protection and/or increases RS or RS metabolites (see e.g. [5,20,36–38] and references therein). However, although reduced antioxidant levels and increased RS levels support the notion that immune system activation is costly, further studies are needed to evaluate whether this results in increased oxidative damage with consequences for survival and fitness.

Blood cells of LPS-challenged lizards exhibited lower total oxidative burst RS production (stamina and strength of the phagocytic response) if their pre-challenge mitochondrial SO levels (measured five days prior to the LPS challenge) were higher. Our results suggest that mitochondrial SO levels may have a regulatory effect on phagocytic RS production. This is consistent with a growing body of research showing that mitochondrial RS levels play an important role in the regulation and fine-tuning of the innate immune response [17,18,39]. With respect to RS-mediated trade-offs, this may suggest that individuals with high inherent RS profiles (i.e. high cellular energy output) are limited in their capacity to mount an immune response due to a higher relative cost of increased RS production through immune system activation [15]. If this is true, it is likely that this trade-off is amplified during periods when heavy investment in other processes (e.g. reproduction) is required (sensu [9]). Notably, Olsson et al. [40] have previously shown that mitochondrial SO levels have relatively high heritability (*h* ^*2*^ = 0.45) and, thus, are a likely target of selection. Given our results, any selection on this component of RS production may also impact on RS production through the immune system.

We found several sex-specific patterns with respect to oxidative burst responses. Male painted dragons showed stronger oxidative burst responses than females after the challenge with LPS, suggesting that this component of the innate immune defense is more robust in males. This result contrasts with our earlier finding that female painted dragons exhibit stronger inflammatory responses towards the mitogen PHA than males [15]. Moreover, the slope of the relationship between the total oxidative burst response and mitochondrial superoxide did not differ between the sexes as might have been expected based on the results from the PHA-challenge experiment (negative relationship between superoxide levels and inflammatory response in males only). However, oxidative burst responses were more strongly related to the current antioxidant status in females than in males. It is possible that the discrepancies between the two studies are due to the fact that the response toward PHA and the oxidative burst response reflect different aspects of immune function. Studies on birds show that a challenge with PHA induces a range of immune responses from both the innate and the acquired immune system [16,41] whereas the oxidative burst response only reflects one (main) aspect of innate immunity [12]. Male and female painted dragons may differently up-regulate the separate parts of the immune system. The sex-specific relationships between oxidative burst responses and plasma antioxidant levels or extracellular RS levels would support such a hypothesis. However, further tests are needed to confirm this.

Immune function is an important fitness trait reflecting the investment in self-maintenance and it is well established that investments in immune function must be traded-off against investments in reproduction [42]. Concepts of ecological immunology assume that sexual dimorphism in immune function is a consequence of life-history differences between the sexes [43]. Male and female painted dragons differ markedly in reproductive tactics with males being more risky and exposed than females (see introduction). It is suggestive that males maximize fitness through an increase in mating rate whereas females maximize fitness through investment in longevity. Hence, one might expect that selection should favour heightened immunity and self-maintenance in females compared to males (Bateman’s principle; e.g. [44]). However, recent empirical and theoretical work suggests that sex differences in immunity are dynamic and may, for example, vary depending on the component of the immune system, the pathogen type or age [43,45]. For example, higher investment in immune function in males may also be expected if the pathogen’s impact on condition is greater for males than females [46].

Although sexual dimorphism in immune function is common in mammals [47,48] with females often having somewhat more robust immune responses than males, evidence for female-biased immune function is less clear for other organism groups [49,50]. For reptiles, there is relatively little information about sex differences in immunity. Several studies report sex differences in immunity, but they often vary with season, context or age [51–56] and, hence, there is currently no clear evidence for stronger immune function in one sex. Interestingly, higher levels of immune system-generated RS in males do not seem to be specific to our study system. A recent study on domesticated turkeys found that heterophil oxidative burst activity was higher in males than in females challenged with *E*. *coli* and *Listeria monocytogenes* bacteria [57].

In summary, our results provide support for the hypothesis that immune system activation is costly, increasing levels of pro-oxidants and reducing levels of antioxidants. Moreover, our results support the idea that mitochondrial SO levels may have an immunoregulatory effect on the oxidative burst response. Such a trade-off may have important consequences for how selection may act on the activity of different RS producing systems, particularly during periods which require heavy investment in other processes. In addition, we found sex differences in oxidative burst activity which may reflect physiological adaptations to sex-specific reproductive strategies. Whether oxidative burst activity is traded off against reproductive investment and how this affects the way RS impact on male and female fitness remain an exciting challenge for future research.

## Supporting Information

S1 Fig Example of oxidative burst reaction curves with and without priming with LPS. The figure depicts response curves from the cell activation assay in which the same lizard was either injected with LPS approximately 18 hours before blood sampling (see main text for injection protocol) or received no injection (samples collected 5 days apart). The x-axis denotes the time course of the assay which lasted 5 min, with one luminescence measurement taken every 5 seconds. Luminescence is expressed in relative luminescence units (RLU) and is proportional to the amount of reactive species produced. Arrows indicate the time points of the automated *in vitro* LPS injections, which resulted in cell activation and elevated production of extracellular reactive species.
(PDF)

S1 File Detailed materials and methods. (PDF)

S1 Tables List of all full models used in the statistical analyses. (PDF)
