# Supplementary figures and images for: Oxidant Trade-Offs in Immunity: An Experimental Test in a Lizard
Source: PLoS One. 2015 May 4;10(5):e0126155. doi: 10.1371/journal.pone.0126155 (PMC4418811; doi:10.1371/journal.pone.0126155)

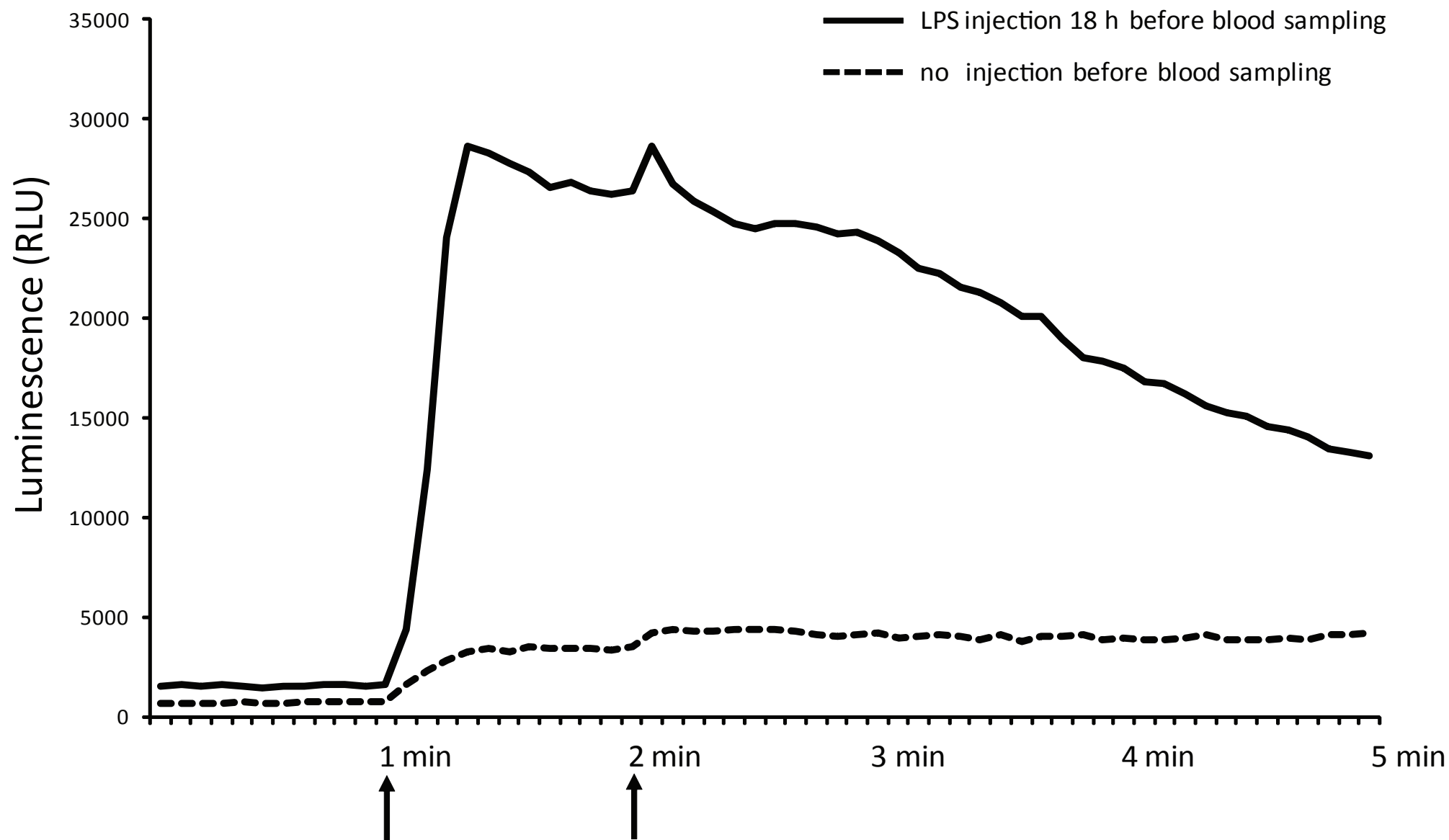

Supplement: S1 Fig — The figure depicts response curves from the cell activation assay in which the same lizard was either injected with LPS approximately 18 hours before blood sampling (see main text for injection protocol) or received no injection (samples collected 5 days apart). The x-axis denotes the time course of the assay which lasted 5 min, with one luminescence measurement taken every 5 seconds. Luminescence is expressed in relative luminescence units (RLU) and is proportional to the amount of reactive species produced. Arrows indicate the time points of the automated in vitro LPS injections, which resulted in cell activation and elevated production of extracellular reactive species. (PDF) [file pone.0126155.s001.pdf]
