# Supplementary material for: Oxidant Trade-Offs in Immunity: An Experimental Test in a Lizard
Source: PLoS One. 2015 May 4;10(5):e0126155. doi: 10.1371/journal.pone.0126155 (PMC4418811; doi:10.1371/journal.pone.0126155)
Supplement: S1 File — (PDF) [file pone.0126155.s002.pdf]

Oxidant trade-offs and the cost of immunity: an experimental test in a lizard

Michael Tobler, Cissy Ballen, Mo Healey, Mark Wilson & Mats Olsson

S1 Supporting information. Because the assays for the quantification of oxidative burst responses and plasma antioxidant levels have not been previously used in any lizard species, we describe them here in more detail.

Phagocytic reactive species production (oxidative burst response)

The production of reactive species (RS) by immune cells constitutes an important part of the innate immune system (Nathan & Cunningham-Bussel 2013). Immune cells such as phagocytes and lymphocytes possess specific enzymes and enzyme complexes (e.g. the nicotinamide adenine dinucleotide phosphate (NADPH) oxidase enzyme complex or the nitric oxide (NO) synthase) that can rapidly produce large amounts of RS (Nathan & Cunningham-Bussel 2013). NADPH oxidase in particular can produce massive amounts of RS within a very short time – this rapid boost of RS is also known as 'oxidative burst' (Halliwell and Gutteridge, 2007).

To estimate the phagocytes' capacity to produce RS, we used the ABEL® cell activation test kit (ABEL-06M; Knight Scientific, Plymouth, UK). This assay makes use of the photoprotein Pholasin®, which becomes chemiluminescent upon reaction with reactive oxygen/nitrogen species, other pro-oxidants (hypochlorous acid, hypobromous acid, bromamine) as well as certain peroxidases (myeloperoxidase, bromoperoxidase). Pholasin® is a 34-kD protein that is too large to enter cells and the light emitted by Pholasin® is, thus, proportional to the amount of extracellular pro-oxidants in a sample. In the ABEL® cell activation assay, Pholasin® is used to monitor rapid changes in pro-oxidant levels in a sample, i.e. the rapid increase of extracellular superoxide in response to activation of the

NADPH oxidase complex (oxidative burst reaction). The oxidative burst reaction can be induced *in vitro* by stimulating blood cells and activating the NADPH oxidase complex with chemical molecules that either bind to extracellular receptors or migrate through the cell-membrane. The ABEL® cell activation test kit includes two such molecules: n-formyl-methionyl-leucyl-phenylalanine (fMLP) for extracellular activation and phorbol-12-myristate-13-acetate (PMA) for intracellular activation. Both of these produce rapid oxidative burst responses in human blood. However, when lizard blood was exposed to these two chemical stimulants, they induced no (fMLP) or only a very small change (PMA) in luminescence. We therefore followed the protocol of Sild and Horak (2010) and used lipopolysaccharide (LPS) from *E.coli* (055:B5; Sigma L2880) to induce the oxidative burst response.

The assay procedure was as follows. Fresh lizard blood was collected in heparinized 0.5 mL eppendorf tubes and refrigerated until analyses (7-10 hours later). Before running the assay, refrigerated blood was incubated for 10 min at 37°C. Subsequently, 5µl of incubated whole blood were diluted in 500µl blood dilution buffer provided with the kit (1:100 dilution). We added 20 µl of diluted whole blood into each of two duplicate sample wells of white luminometer plates pre-filled with 85 µl PBS, 20 µl of reconstituted Adjuvant-K™ and 50 µl of the photoprotein Pholasin® (10µg mL^{-1}). Initial measurements indicated that the oxidative burst response was relatively slow (reaching a maximum after about 20-30 sec) compared to responses reported in birds which peak after a few seconds (Sild and Horak 2010). To speed up sample processing, we therefore adjusted the protocol running three duplicate samples (6 wells) at the same time with measurement intervals (cycles) of 5 seconds instead of 1 second. Plates loaded with samples were placed into a luminescence microplate reader (LUMIstar Optima, BMG Labtech, Mornington, Australia). The incubator of the LUMIstar was set to 37°C and before the start of measurements, plates were incubated again for 5 min. Each run lasted 5 min (60 cycles). One injector of the LUMIstar was primed with LPS (1 mg mL^{-1} PBS) and set to inject 25 µl after 1 min (cycle 12) and 2 min (cycle 24). The rationale of

having two injections was to make use of a priming effect of the first injection and thereby induce a stronger secondary oxidative burst response after the second injection (see Sild & Horak 2010). Although comparisons with samples that were run concomitantly and only received one LPS-injection showed that the second injection increased luminescence values by 5-25%, the secondary injection did not induce a prominent secondary burst reaction (see also Fig. 1). Moreover, later validation showed that parameter values of samples that were injected once or twice, respectively, were strongly correlated ($r_{15}>0.85$, $p<0.0001$ in all cases). We therefore did not calculate separate response variables for the intervals before and after the second injection. Instead, we calculated the following parameters from the mean values of the duplicate samples: the peak luminescence (*peak oxidative burst response*) occurring after the first injection with LPS and the cumulative luminescence (*total oxidative burst response*) from the first injection until the end of the assay run (cycles 13-60). The peak oxidative burst response is a measure of the strength of the phagocytic response (the maximum capacity of the phagocytes to produce RS in response to stimulation) whereas the total oxidative burst response is a measure of both the strength and the stamina of the phagocytic response (the total amount of RS produced in response to stimulation). In addition to these two parameters, we also calculated the average luminescence prior to the first injection (cycle 1-12). This is an estimate of the *baseline extracellular RS* produced by the blood cells at the time of measurement. The repeatabilities (Lessells and Boag, 1987) of mean values from duplicate samples run on the same day were R=0.74 ($F_{12,13}=6.67$, p=0.0009), R=0.91 ($F_{12,13}=23.72$, p<0.0001) and R=0.86 ($F_{12,13}=12.95$, p<0.0001) for baseline extracellular RS, peak and total oxidative burst response, respectively. We found no evidence that these measures were affected by a time lag from blood sampling to measurement such as reported in Sild and Horak (2010) (-0.12<r<0.21, p>0.15 for all measures). To account for differences in phagocytic RS measures between the sampling batches which can be attributed to variation in assay conditions (mainly due to variation in Pholasin® and Adjuvant-K^{TM} reactivity) we

standardised the data by batch, setting means to zero and standard deviations to one before pooling the data for analysis. We also included wells without Pholasin® and/or without blood cells as controls. Controls never showed elevated luminescence in response to LPS.

Quantification of plasma antioxidant levels

Lizard blood not used in flow cytometry and cell activation assays was centrifuged at 1000 ***g*** and plasma was stored at –80 ºC until later analysis. Plasma antioxidant status was measured using a commercial test kit (Abel®-41M2) purchased from Knight Scientific Limited (Plymouth, UK). Abel®-41M2 is a chemiluminescent test which allows the assessment of the total antioxidant status. The test measures the capacity of a plasma sample to withstand oxidation by peroxynitrite, which has high oxidant potential and occurs naturally in inflammatory cells, such as neutrophils and macrophages.

The basic principle of the test is as follows: The test uses the photoprotein Pholasin® which emits light in the presence of reactive oxygen and nitrogen species and peroxidase enzymes (see above). Antioxidants in the plasma sample (3 μl) compete with Pholasin® for the reactive oxygen species peroxynitrite ($ONOO^-$) which is produced in the assay by the reaction between superoxide and nitric oxide, released simultaneously and continually from a 2.5 mmol L^{-1} solution of 3-morpholino-sydnonimine HCl ($C_6H_{10}N_4O_2 \cdot HCl$; SIN-1). In the absence of other antioxidants, Pholasin® emits light with gradually increasing intensity, reaching a peak after a few minutes. If there are antioxidants in the sample, they will compete with the Pholasin® for the peroxynitrite and this will delay the time at which the peak luminescence occurs. The more peak luminescence is delayed, the higher is the total antioxidant capacity of the test sample. Time until peak luminescence (i.e. total antioxidant capacity) for individual samples was standardised by reference to a serially diluted standard (vitamin E analogue (VEA) 6-hydroxy-2,5,7,8-tetramethyl-chroman-2-carboxylic acid) which

was included on each plate. Total antioxidant capacity is expressed in VEA equivalent units $\mu molL^{-1}$ (standard range: 50-800 $\mu molL^{-1}$) which are derived from the linear regression formula of the standard curve. We performed the assay according to the manufacturer's instructions but with slightly reduced reagent and sample volumes. In each well, we added 57 µl of assay buffer, 30 µl of Pholasin® and 3 µl of lizard plasma. The plate was placed into a luminescence microplate reader (LUMIstar Optima) with the incubator set at 30°C. At the start of the assay, the plate was shaken for 4 s (shaking frequency 500 r.p.m., double orbital) and 30 µl SIN-1 were injected with an automatic dispenser into each well (cycle 1). Cycle time was 51 sec and each plate was run for at least 38 cycles to ensure that peak luminescence would fall within assay time. All samples were run in duplicates. The mean of the duplicate values expressed in standard VEA equivalent units $\mu molL^{-1}$ was used in the statistical analyses (see below). Interassay repeatability for antioxidant levels (duplicate samples run on different plates, but on the same day) was R=0.89 ($F_{19,20}=16.58$, $p<0.0001$). One plate with samples from the first sampling event was erroneously run with the incubator set at 37°C, which resulted in shorter time to peak (i.e. lower VEA units). As we had not enough plasma to re-run all the samples and because later validation showed that VEA units obtained from assays run at 30°C or 37°C are highly correlated ($r_{16}=0.96$, $p<0.0001$), we instead standardised the data by assay run (30°C or 37°C), setting the means to zero and standard deviations to one before pooling the data from the samples run at different temperatures. When analysing the data, we found also found a significant difference between batches from the second sampling event which we deduced were due to different batches of reconstituted Pholasin®. Hence, these data were also standardised as above.

References

Halliwell, B., Gutteridge, J., 2007. Free Radicals in Biology and Medicine. Oxford University Press, Oxford UK.

Lessells, C. & Boag, P. T. (1987) Unrepeatable repeatabilities: a common mistake. *The Auk,* **104**, 116-121.

Nathan, C., Cunningham-Bussel, A., 2013. Beyond oxidative stress: an immunologist's guide to reactive oxygen species. Nat. Rev. Immunol. **13**, 349–361.

Sild, E. & Hõrak, P. (2010) Assessment of oxidative burst in avian whole blood samples: validation and application of a chemiluminescence method based on Pholasin. *Behavioral Ecology and Sociobiology*, **64**, 2065-2076.
