# Supplementary material for: Oxidant Trade-Offs in Immunity: An Experimental Test in a Lizard
Source: PLoS One. 2015 May 4;10(5):e0126155. doi: 10.1371/journal.pone.0126155 (PMC4418811; doi:10.1371/journal.pone.0126155)
Supplement: S1 Tables — (PDF) [file pone.0126155.s003.pdf]

Oxidant trade-offs and the cost of immunity: an experimental test in a lizard

Michael Tobler, Cissy Ballen, Mo Healey, Mark Wilson & Mats Olsson

S1 Tables. List of all full models used in the statistical analyses.

Table 1. Full model assessing the effect of sex and four baseline estimates of oxidative status on *peak* oxidative burst response. A likelihood ratio test of the full model against a null model (with intercept only) yielded χ^2=13.7, d.f.=9, p=0.13. Abbreviations: mSO=mitochondrial superoxide; iRS=unspecific intracellular reactive species; eRS=unspecific extracellular reactive species; plasma AOL=plasma antioxidant levels.

	d.f.	F-value	P-value
Sex	1,50	0.06	0.80
mSO	1,50	0.31	0.58
mSO×sex	1,50	0.02	0.90
iRS	1,50	0.97	0.33
iRS×sex	1,50	0.30	0.59
eRS	1,50	15.08	0.0003
eRS×sex	1,50	0.08	0.79
plasma AOL	1,50	0.53	0.47
plasma AOL×sex	1,50	0.00	0.96

Table 2. Full model assessing the effect of sex and four baseline estimates of oxidative status on *total* oxidative burst response. Likelihood ratio test of full model against null model: χ^2=17.5, d.f.=9, p=0.041. Abbreviations: see Table 1.

	d.f.	F-value	P-value
Sex	1,50	0.06	0.81
mSO	1,50	0.90	0.35
mSO×sex	1,50	0.35	0.56
iRS	1,50	0.06	0.80
iRS×sex	1,50	1.57	0.22
eRS	1,50	15.46	0.0003
eRS×sex	1,50	0.01	0.93
plasma AOL	1,50	2.01	0.16
plasma AOL×sex	1,50	0.11	0.75

Table 3. Full model assessing the effect of sex and LPS-treatment on eRS. Likelihood ratio test of full model against null model: χ^2=60.7, d.f.=6, p<0.0001. Abbreviations: see Table 1.

	d.f.	F-value	P-value
Sex	1,53	0.63	0.43
Treatment	1,53	116.35	<0.0001
Treatment×sex	1,53	2.78	0.10
Pre-challenge eRS	1,53	0.60	0.44
Pre-challenge eRS×sex	1,53	0.04	0.84
Pre-challenge eRS × treatment	1,53	0.13	0.72

Table 4. Full model assessing the effect of sex and LPS treatment on plasma AOL.

Likelihood ratio test of full model against null model: χ^2=22.9, d.f.=6, p=0.0008.

Abbreviations: see Table 1.

	d.f.	F-value	P-value
Sex	1,54	0.68	0.41
Treatment	1,54	27.75	<0.0001
Treatment×sex	1,54	0.89	0.35
Pre-challenge plasma AOL	1,54	4.78	0.033
Pre-challenge plasma AOL×sex	1,54	0.01	0.91
Pre-challenge plasma AOL × treatment	1,54	1.04	0.31

Table 5. Full model assessing the effect of sex and LPS treatment on the *peak* OBR.

Likelihood ratio test of full model against null model: χ^2=140.1, d.f.=9, p<0.0001.

Abbreviations: see Table 1.

	d.f.	F-value	P-value
sex	1,50	4.57	0.037
treatment	1,50	63.39	<0.0001
treatment×sex	1,50	0.03	0.86
pre-challenge peak OBR	1,50	9.62	0.003
pre-challenge peak OBR×sex	1,50	0.56	0.46
pre-challenge peak OBR×treatment	1,50	0.92	0.34
eRS	1,50	36.36	<0.0001
eRS×sex	1,50	0.04	0.84
eRS× treatment	1,50	2.47	0.12

Table 6. Full model assessing the effect of sex and LPS treatment on the *total* OBR. Likelihood ratio test of full model against null model: χ^2=127.9, d.f.=9, p<0.0001. Abbreviations: see Table 1.

	d.f.	F-value	P-value
sex	1,50	4.52	0.038
treatment	1,50	44.27	<0.0001
treatment×sex	1,50	0.05	0.83
Pre-challenge total OBR	1,50	7.40	0.009
Pre-challenge total OBR×sex	1,50	0.03	0.87
Pre-challenge total OBR ×treatment	1,50	0.01	0.92
eRS	1,50	33.74	<0.0001
eRS×sex	1,50	0.33	0.57
eRS×treatment	1,50	2.04	0.16

Table 7. Full model assessing whether pre-challenge peak OBR, eRS and plasma AOL predicts post-challenge *peak* OBR. Likelihood ratio test of full model against null model: χ^2=21.1, d.f.=7, p=0.004. Abbreviations: see Table 1.

	d.f.	F-value	P-value
sex	1,21	5.21	0.033
eRS	1,21	14.62	0.001
eRS×sex	1,21	0.23	0.63
plasma AOL	1,21	2.46	0.13
plasma AOL×sex	1,21	2.75	0.11
pre-challenge peak OBR	1,21	0.86	0.37
pre-challenge peak OBR×sex	1,21	0.57	0.46

Table 8. Full model assessing whether pre-challenge total OBR, eRS and plasma AOL can predict post-challenge *total* OBR. Likelihood ratio test of full model against null model: χ^2=20.8, d.f.=7, p=0.0041. Abbreviations: see Table 1.

	d.f.	F-value	P-value
sex	1,21	6.28	0.021
eRS	1,21	10.99	0.003
eRS×sex	1,21	0.75	0.40
plasma AOL	1,21	1.64	0.21
plasma AOL × sex	1,21	3.04	0.10
pre-challenge total OBR	1,21	1.15	0.30
pre-challenge total OBR×sex	1,21	0.01	0.92

Table 9. Full model using pre-challenge baseline estimates of RS as predictor variables for *peak* OBR. Likelihood ratio test of full model against null model: χ^2=20.1, d.f.=9, p=0.018. Abbreviations: see Table 1.

	d.f.	F-value	P-value
sex	1,19	3.32	0.08
mSO	1,19	1.59	0.22
mSO×sex	1,19	0.76	0.39
iRS	1,19	0.36	0.55
iRS×sex	1,19	0.42	0.53
eRs	1,19	8.17	0.01
eRS×sex	1,19	2.48	0.13
pre-challenge total OBR	1,19	1.15	0.30
pre-challenge total OBR×sex	1,19	0.45	0.51

Table 10. Full model using pre-challenge baseline estimates of RS as predictor variables for *total* OBR. Likelihood ratio test of full model against null model: χ^2=23.0, d.f.=9, p=0.006. Abbreviations: see Table 1.

	d.f.	F-value	P-value
sex	1,19	5.26	0.034
mSO	1,19	5.23	0.034
mSO×sex	1,19	1.45	0.24
iRS	1,19	0.13	0.72
iRS×sex	1,19	0.11	0.75
eRs	1,19	6.32	0.021
eRS×sex	1,19	7.15	0.015
pre-challenge total OBR	1,19	2.05	0.17
pre-challenge total OBR×sex	1,19	0.08	0.77
